# Injury of the Radial Nerve in the Arm: A Review

**DOI:** 10.7759/cureus.2199

**Published:** 2018-02-16

**Authors:** Taroob J Latef, Muhammad Bilal, Marc Vetter, Joe Iwanaga, Rod J Oskouian, R. Shane Tubbs

**Affiliations:** 1 Research, Seattle Science Foundation; 2 Department of Medicine, Dow University of Health Sciences (DUHS), Karachi, Pakistan; 3 Seattle Science Foundation; 4 Neurosurgery, Swedish Neuroscience Institute; 5 Neurosurgery, Seattle Science Foundation

**Keywords:** neuropathy, brachial plexus, anatomy, compression, upper limb

## Abstract

Compression of the radial nerve is most commonly described at the supinator muscle (i.e., arcade of Frohse). However, radial nerve compression can occur in the arm. Therefore, the purpose of this article is to review both etiologies of radial nerve entrapment and the sites at which this can occur in the arm. The clinical presentation of radial nerve entrapment in the arm and how it differs from that of entrapment at other sites is reviewed and the conditions potentially predisposing to nerve entrapment are described. Particular attention is paid to the nerve’s course and potential variants of the anatomical structures in the arm. In each case, the recommended course of management for the neuropathy is described. Injury of the radial nerve can arise from a varied set of pathologies including trauma, tumors, anomalous muscles, and intramuscular injections. Physicians should have a good working knowledge of the anatomy and potential mechanisms for radial nerve injury.

## Introduction and background

Radial neuropathy can be divided into compressive and non-compressive neuropathies [1]. Although injury to the nerve can occur anywhere along its course, compressive neuropathy of the radial nerve results from entrapment of the nerve at specific locations. These are sites where the nerve lies superficially, penetrates muscles, or travels through narrow bony canals [1,2]. The radial nerve is prone to entrapment at three different sites: as it passes between the heads of the triceps brachii muscle [3], in the spiral groove of the humerus [1], and while piercing the lateral intermuscular septum [3]. Compression neuropathy can cause nerve injury from direct pressure or pressure-induced ischemia. The nerve injuries can be graded according to severity as neuropraxia, axonotmesis, and neurotmesis. The degree of the injury depends on the force and duration of compression. Neuropraxia is a transient interruption in the transmission of electrical signals with spontaneous recovery. Axonotmesis is more severe, with direct damage to the axons resulting in Wallerian degeneration and late recovery. Neurotmesis is the complete destruction of axons and Schwann cells with no chance of recovery without surgery [2-4].

Anatomy

**Figure 1:**
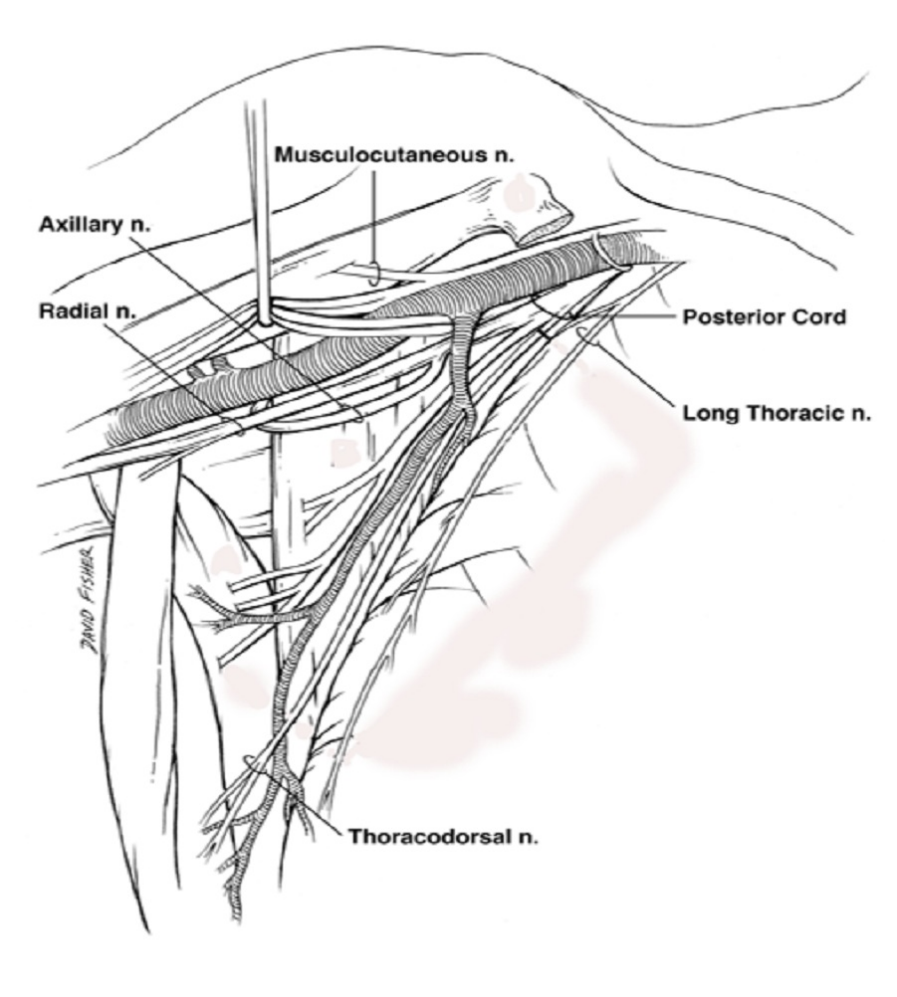
Schematic view of the axillae illustrating the proximal radial nerve coursing from its origin from the posterior cord of the brachial plexus to the proximal arm.

**Figure 2:**
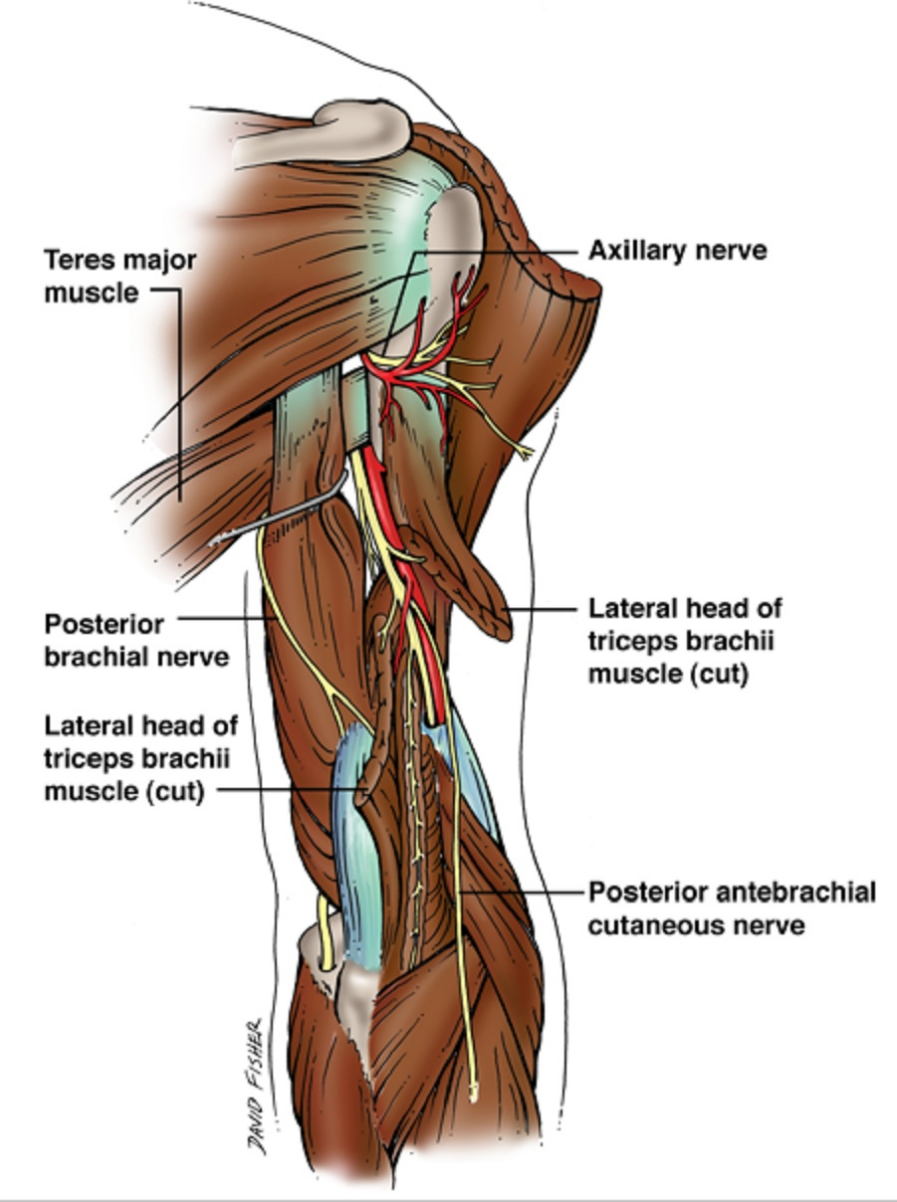
Schematic drawing of the radial nerve as it courses in the posterior arm. Also note the cutaneous branches of the nerve.

**Figure 3:**
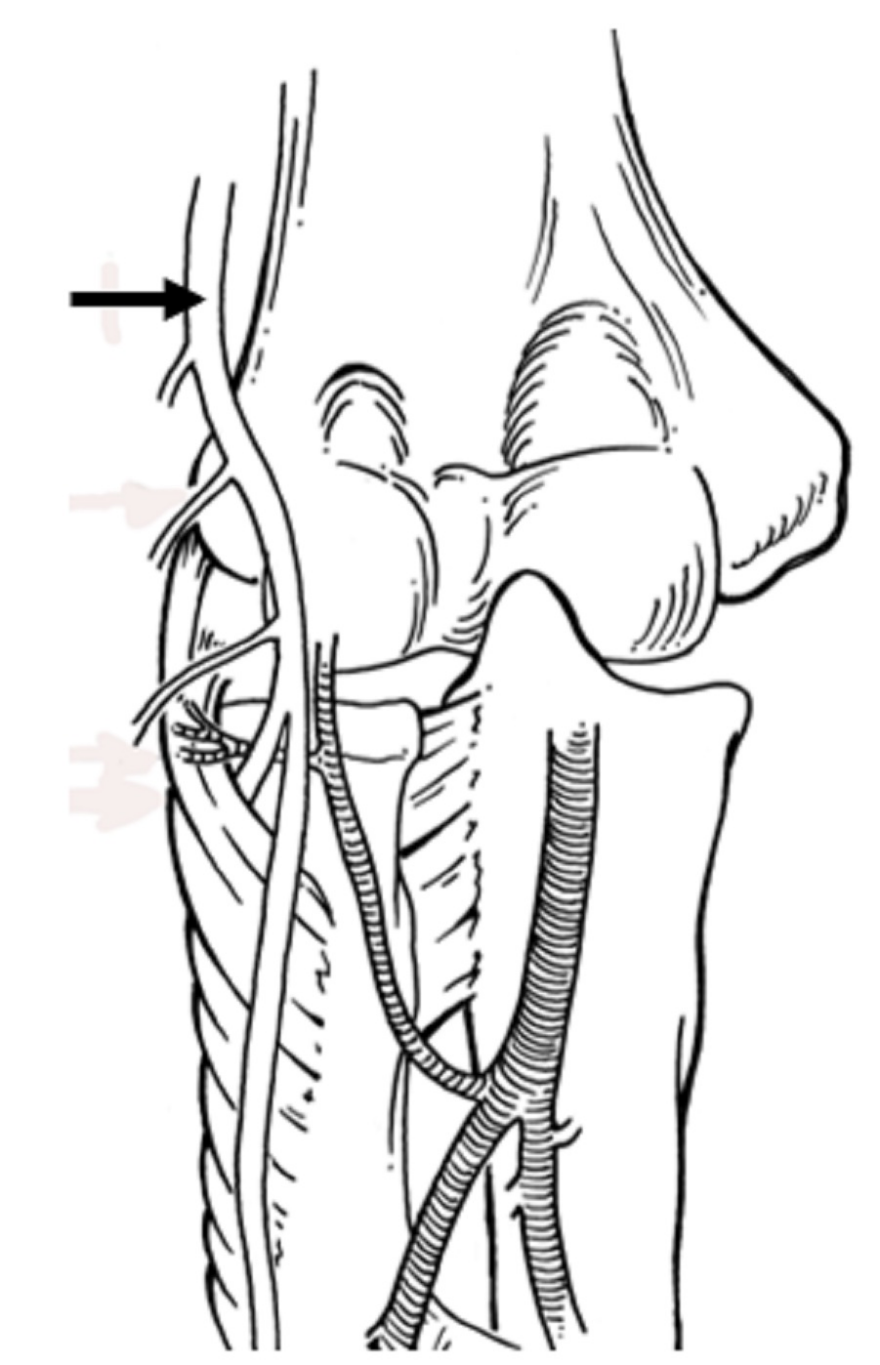
Schematic drawing of the distal arm and the course of the radial nerve (arrow) anterior to the lateral epicondyle.

The anatomy of the radial nerve is clinically significant, and the structures along its course play an important role in determining the sites at which entrapment neuropathy might occur [5]. After originating from the brachial plexus (Figure 1), the radial nerve traverses the triangular interval at the inferior aspect of the teres major muscle and enters the posterior compartment of the arm (Figure 2) [3]. It courses between the lateral and medial heads of the triceps brachii muscle in the proximal two-thirds of the humerus. In the distal third, it travels directly in the spiral groove of the humeral shaft. The spiral groove is located between the lateral and posterior aspect of the humeral diaphysis. The radial nerve then pierces the lateral intermuscular septum to enter the anterior compartment of the arm (Figure 3). It courses between the brachialis and brachioradialis muscles before entering the forearm [1,3]. The spiral groove, also known as the radial groove [5], and the intermuscular septum are important landmarks in relation to the radial nerve in the arm [6].

## Review

Etiology

Humeral Shaft Fracture

Humeral shaft fractures are the leading cause of radial neuropathy in the arm [3]. These are commonly spiral fractures between the middle and distal thirds of the humeral shaft. A spiral fracture in the distal third followed by proximal and radial displacement of the distal bone fragment is also known as a Holstein-Lewis fracture [7]. Unlike the proximal third of the humerus, where the radial nerve is separated from bone by triceps brachii muscle fibers, in the distal third of the arm, the radial nerve lies directly over the periosteum with no interposed muscle. Here, the nerve also has greatly restricted mobility as it pierces the lateral intermuscular septum. The radial nerve is most susceptible to injury in the distal one-third of the arm [3,7]. It can be compressed between the overlapping bone fragments leading to entrapment neuropathy. In a study conducted to assess locked nailing of spiral humeral fractures, the authors concluded that the risk of radial nerve entrapment was considerably high in fractures from high impact trauma and those causing varus angulations [8].

Secondary to Lateral Intermuscular Septum Compression

The lateral intermuscular septum is also a common site of radial nerve compression. A known site of limited mobility for the radial nerve, the lateral intermuscular septum can cause chronic compression after an insult to the humerus. In one reported case, delayed radial neuropathy occurred three months after a humeral shaft fracture and was the result of the radial nerve’s entrapment within the lateral intermuscular septum [9].

Secondary to Callus Formation

The radial nerve can become encased within a healing callus of the fractured humeral shaft. In such cases, symptoms manifest gradually as the callus forms, requiring surgical removal of the callus to restore nerve function [10]. In one case, a patient presented with delayed radial neuropathy nine months [11] after a diaphyseal fracture of the humerus and in another case, three years later [12]. During surgical exploration, the radial nerve was found to be compressed at the callus site in both patients. The neurological function returned gradually after intraoperative decompression of the radial nerve [11,12]. Hence, patients suffering from humeral fractures with no neurological deficits at the time of presentation can still develop delayed radial nerve palsy over a period of months to years [10].

Secondary to Fracture Manipulation

A “secondary radial palsy” during fracture manipulation or reduction can occur as a result of intraoperative nerve exploration or the surgical approach used [13]. The radial nerve is extremely sensitive to even slight tension exerted during surgical exploration of the fracture. The position of the patient during general anesthesia is also a causative factor as loss of muscle tone can lead to inadvertent traction at the fracture site and compression of the radial nerve [13]. For the most part, radial nerve entrapment is seen after fracture manipulation when the nerve is unknowingly entrapped between bone and an installed plate, compressed by a bone fragment or if excessive nailing of the bone occurs [14]. A study concluded that in 78% of patients suffering from iatrogenic radial nerve palsy, the injury occurred after a plate osteosynthesis approach. In many of these patients, the nerve was explored during acute fracture management and found to be intact, confirming the use of this surgical approach as the cause of radial nerve entrapment [13].

Compression by Lateral Head of Triceps Brachii

Compression of the radial nerve can also be caused by the fibrous arch of the lateral head of the triceps muscle in the arm [3]. Following strenuous muscular effort at the elbow, some individuals were reported to have suffered from radial nerve paralysis. Through electrophysiological examination, the lesion was found to be present at the site where the radial nerve gives off branches to the triceps muscle. This is the point where the radial nerve courses through the posterior compartment of the arm between the lateral and medial heads of the triceps muscle and consistently passes through a fibrous arch [15]. The fibrous arch consists of muscle fibers that originate from the tendon of the lateral head of the triceps muscle and insert just below the lateral part of the spiral groove [16]. Thus, apart from its origin from the humerus, the lateral head of the triceps also receives fibers from this arch. This fibrous arch may be tight in some individuals, providing a restricted passage for the radial nerve and leading to compression during extensive muscular effort when increased blood flow causes the triceps to swell [15]. In most cases, compression by the fibrous arch results in neuropraxia [15]. However, cases have been reported where relief of symptoms did not occur and surgical exploration was necessary [17].

Tumors

Radial nerve compression in the arm can also occur from a tumor growth. It can be either a malignant soft tissue mass causing compression of the nerve through infiltration [18] or a benign growth in a closed anatomical space [19] (such as the posterior or anterior compartments of the arm).

Blood Pressure Cuff

Perioperative radial nerve compression can result from prolonged inflation of an automatic blood pressure cuff around the arm, especially in a lean patient [20]. One of the main reasons this type of compression occurs is because the blood pressure cuff is placed over the distal third of the arm. In this third, the radial nerve lies in direct contact with the humerus and there are no muscle fibers to act as a cushion between the nerve and the periosteum of the bone [20].

Intramuscular Injections

Radial nerve entrapment can occur as a delayed complication of a chronic intramuscular injection [21]. The intramuscular injection leads to muscle fibrosis at the injected site, which can be proximal to the spiral groove of the humerus or include the triceps brachii muscle [3]. Exploration in such cases has found the radial nerve entrapped within densely fibrotic muscle fibers [21].

Anomalous Brachioradialis Muscle

A rare variation of the brachioradialis muscle, which originates from the acromion instead of the lateral supracondylar ridge of the humerus and blends with the normal brachioradialis muscle, has been known to cause compression of the radial nerve in the arm [22]. This occurs when the variant muscle crosses over the radial nerve in the anterior compartment of the arm creating a narrow tunnel between its fibers [23] or with those of the biceps brachii muscle [22].

Saturday Night Palsy

The term “Saturday Night Palsy” is used for a radial nerve injury caused by prolonged compression of the nerve at the spiral groove. The origin of the term is due to the association of the condition with a night spent in alcoholic stupor with the arm draped over a chair or bench [3,4,24]. Mechanical compression of the radial nerve in the spiral groove can also occur as a result of the continuous use of crutches or prolonged kneeling in a “shooting” position [4]. 

Clinical presentation

Entrapment of the radial nerve in the arm can cause a myriad of signs and symptoms, the most often diagnosed of which includes loss of the ability to supinate the forearm while still being able to extend it. Loss of forearm extension can occur when the radial nerve is injured in the axilla. Injury to the nerve in the arm does not significantly impact the triceps and the anconeus, therefore the ability to extend the forearm remains intact [24]. Motor deficits include loss of mobility in the brachioradialis and supinator muscle, which supinate the forearm, as well as loss of mobility in the extensor carpi radialis longus and brevis, which extends the wrist. An inability to extend the fingers at the metacarpophalangeal joints may also result. Extension of the interphalangeal joints remains intact as the lumbricals and interossei are innervated by the ulnar nerve [3]. The sensory deficits include loss of sensation in the posterior forearm, dorsum of the hand and lateral three and a half fingers. Sensation in the posterior arm remains intact as it is innervated by the radial nerve before it reaches the diaphysis of the humerus [6]. These signs are often accompanied with pain, tingling, and numbness [10].

Management

Radial nerve palsy can be classified into complete or partial and primary or secondary [25]. Radial nerve entrapment syndromes are generally transient and are treated conservatively with nonsteroidal anti-inflammatory medications, corticosteroids, and rest. Maintenance of a full passive range of motion is critical during therapy [3]. Many nondisplaced humeral fractures are also treated similarly and kept under observation [25]. Surgical exploration is recommended in the presence of an open fracture, high energy trauma, a compressive lesion, or the failure of conservative treatment [26]. The prognosis for radial entrapment neuropathies depends on the extent of radial nerve injury. With neuropraxia, recovery is complete in all cases. In some cases, axonotmesis and neurotmesis can also show signs of recovery but most often require surgical exploration.

## Conclusions

Physicians should have a good working knowledge of the anatomy and potential mechanisms for radial nerve injury in the arm. A thorough physical examination can help isolate the site of the nerve lesion.
